# Prevalence and risk factors of *Eimeria* spp. natural infection in sheep from northern Paraná, Brazil

**DOI:** 10.1590/S1984-29612022004

**Published:** 2022-01-05

**Authors:** Priscilla Gomes Carneiro, João Pedro Sasse, Ana Clécia dos Santos Silva, Mércia de Seixas, Aline Ticiani Pereira Paschoal, Ana Flávia Minutti, Thais Agostinho Martins, Sérgio Tosi Cardim, Fernando de Souza Rodrigues, Luiz Daniel de Barros, João Luis Garcia

**Affiliations:** 1 Laboratório de Protozoologia Animal, Departamento de Medicina Veterinária Preventiva, Universidade Estadual de Londrina – UEL, Londrina, PR, Brasil; 2 Departamento de Medicina Veterinária, Universidade Norte do Paraná – UNOPAR, Arapongas, PR, Brasil

**Keywords:** Risk factors, epidemiology, coccidiosis, sheep, Fatores de risco, epidemiologia, coccidiose, ovinos

## Abstract

The present study aimed to perform an epidemiological and morphological identification of *Eimeria* infection in sheep in Brazil. Fecal samples from sheep were collected from 20 farms in northern Paraná, Brazil. An epidemiological questionnaire was used to evaluate the risk factors. Fecal samples containing oocysts per gram of feces (OoPG) ≥1000 were subjected to the modified Willis-Mollay method to perform oocyst identification. Sporulated oocysts were observed microscopically for morphological identification. A total of 807 fecal samples were collected. Based on the morphological characteristics of the sporulated oocysts, 10 species of *Eimeria* were identified, with main species observed: *Eimeira ovinoidalis* (98.1%), *Eimeria crandallis* (87.6%), *Eimeria parva* (79.1%), and *Eimeria bakuensis* (60.8%). Only 2.6% (7/268) of the sheep were infected with a single species, 4.8% (13/268) contained two different species, and 92.5% (248/268) were infected with three or more species. The analysis of risk factors showed that an intensive rearing, no rotation of pasture, dirt, and slatted floors, and age up to 12 months were associated with infection. This study showed a high prevalence of *Eimeria* natural infection in sheep from northern Paraná, Brazil. Furthermore, based on the risk factors, good management and hygiene practices must be employed to avoid infection.

## Resumo

O presente estudo teve como objetivo realizar uma avaliação epidemiológica e morfométrica da infecção por *Eimeria* em ovinos no Brasil. Amostras fecais de ovinos foram coletadas em 20 fazendas no sul do Brasil. Um questionário epidemiológico foi utilizado para avaliar os fatores de risco. Amostras fecais, contendo oocistos por grama de fezes (OoPG) ≥1000, foram submetidas ao método de Willis-Mollay modificado para realizar a identificação de oocistos. Oocistos esporulados foram observados microscopicamente para identificação morfológica. Foram coletadas 807 amostras fecais. Com base nas características morfológicas e morfométricas dos oocistos esporulados, foram identificadas 10 espécies de *Eimeria*, com as principais espécies observadas: *Eimeria ovinoidalis* (98,1%), *Eimeria crandallis* (87,6%), *Eimeria parva* (79,1%) e *Eimeria bakuensis* (60,8%). Apenas 2,6% (7/268) dos ovinos estavam infectados com uma única espécie, 4,8% (13/268) continham duas espécies diferentes e 92,5% (248/268) estavam infectados com três ou mais espécies. A análise dos fatores de risco mostrou que uma criação intensiva, sem rotação de pasto, terra, piso de ripa e idade até 12 meses foram associadas à infecção. Este estudo mostrou uma alta prevalência de infecção natural por *Eimeria* em ovinos do norte do Paraná, Brasil. Além disso, com base nos fatores de risco, boas práticas de manejo e higiene devem ser empregadas para evitar infecções.

## Introduction

Coccidiosis is an intestinal disease caused by obligate intracellular protozoans of the genus *Eimeria* that infects different animal species, including birds, ruminants, and rabbits (Bruhn et al., 2011; Cardim et al., 2018; Mohamaden et al., 2018; Basiaga et al., 2020). In sheep, the infection causes high economic impact due to clinical and subclinical disease, which results in decreased productivity and low growth of infected animals (Chartier & Paraud, 2012). Farmers usually underestimate subclinical coccidiosis; however, low productivity accounts for more economic losses than mortality, which rarely exceeds 10% of the infected herd (Silva et al., 2007; Chartier & Paraud, 2012).

Although different *Eimeria* species can infect sheep, not all of them are associated with the disease. *Eimeria ovinoidalis*, *E. crandallis*, *E. bakuensis*, *E. parva*, and *E. ahsata* are the main pathogenic species; however, mixed infections are more common in naturally infected animals (Arslan et al., 1999; Keeton & Navarre, 2018). Morphological studies have shown that the distribution of *Eimeria* species have regional variations, and that *E. crandallis* and *E. ovinoidalis* are the main species affecting sheep in Brazil (Lopes et al., 2013; Souza et al., 2015).

Given the problems caused by eimeriosis, ascertaining the distribution of different species is essential to develop and adopt more effective control strategies. Thus, this study aimed to conduct an epidemiological and morphological study of *Eimeria* in sheep from northern Paraná, Brazil.

## Material and Methods

### Animals and study area

**Figure 1:**
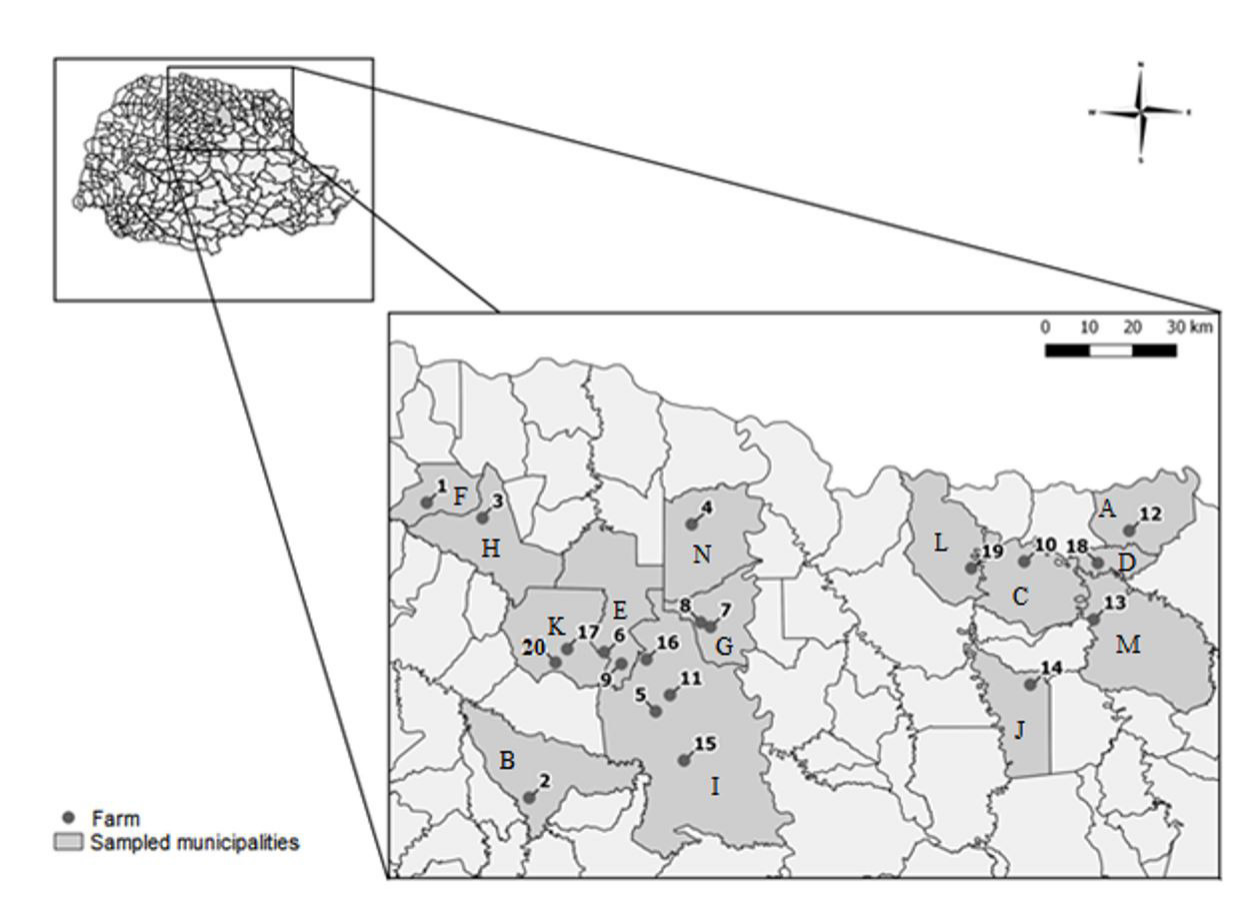
Map showing the farms sampled from northern Paraná, Brazil.Municipalites: (A) Cambará; (B) Apucarana; (C) Bandeirantes; (D) Barra do Jacaré; (E) Cambé; (F) Guaraci; (G) Ibiporã; (H) Jaguapitã; (I) Londrina; (J) Ribeirão do Pinhal; (K) Rolândia; (L) Santa Mariana; (M) Santo Antônio da Platina; (N) Sertanópolis.

Fecal samples were collected randomly and directly from the rectal ampoule of sheep of both sexes and different ages between November 2016 and September 2018 from 20 farms in 14 municipalities (Cambará, Apucarana, Bandeirantes, Barra do Jacaré, Cambé, Guaraci, Ibiporã, Jaguapitã, Londrina, Ribeirão do Pinhal, Rolândia, Santa Mariana, Santo Antônio da Platina, and Sertanópolis) in the northern region of the state of Paraná, southern Brazil (Figure 1). During this period, the maximum and minimum temperature averages were 28.6 °C and 18.4 °C, respectively. The average precipitation was 2.2 mm per day, and the relative humidity was 73% (IAPAR, 2018). The sample size was calculated using the OpenEpi software v. 3.01, assuming a prevalence of 50%, absolute precision of 5%, confidence level of 95%, and a population of 18,696 animals. An epidemiological questionnaire was applied to all farmers to evaluate risk factors (age, sex, production system, rotation of pasture, and type of facilities) associated with infection. All procedures involving animals in this study were approved by the Animal Ethics Committee of the State University of Londrina (CEUA n. 182/2015).

### Coproparasitological examination and morphological identification

Quantification of *Eimeria* oocysts quantification was performed using the Gordon and Whitlock technique, as previously described (Ueno & Gonçalves, 1998). Samples with oocysts per gram of feces (OoPG) ≥ 1,000 were subjected to the Willis-Mollay technique (Ueno & Gonçalves, 1998) for oocyst identification. The feces were diluted in an aqueous potassium dichromate (K_2_Cr_2_O_7_) solution at a final concentration of 2.5%. This solution was distributed in Petri dishes and incubated at 25 °C for 10 days or at least 70% of sporulated oocysts, as described by Florião et al. (2016). After sporulation, samples were resubjected to the Willis-Mollay technique, and the sporulated oocysts were observed microscopically for morphological identification according to previously described parameters (Fallis, 1974; Eckert et al., 1995). Species identification was performed using an Olympus BX43 optical microscope (Olympus Corporation, Tokyo, Japan) at 100 × magnification attached to a digital camera (Q-Color3™ Imagim System, Olympus Corporation) for oocyst photo-documentation. The digital images were analyzed using the CellSens Standard software, version 1.15 2016 (Olympus Corporation).

### Statistical analysis

All variables were analyzed using the chi-squared test (χ^2^) corrected by Yates, and the *odds ratio* (OR) was calculated as the measure of association. Statistical significance was set at P ≤0.05. Statistical analysis was performed using EpiInfo7 version 7.2.2.6 and OpenEpi version 3.01 software.

## Results

In total, 807 fecal samples were collected, of which 176 were from male, and 631 were from female animals. Of the 807 samples, 662 (82.03%) were positive for *Eimeria* spp., and 268 (40.48%, 268/662) had OoPG > 1000. Microscopic analyses revealed 10 species, with *E. ovinoidallis* being the most prevalent, followed by *E. crandallis* and *E. parva*. Table 1 shows the prevalence of all identified species. Most of the animals (92.54%, 248/268) were infected with three or more species, while 7 (2.61%) and 13 (4.85%) were infected with only one and two species, respectively.

**Table 1:** Prevalence of *Eimeria* species in sheep from northern Paraná, Brazil.

***Eimeria* Species**	**% of positive samples (n/total)** *	**CI 95%** **	**% of positive farms (n/total)**
*E. ovinoidalis*	98.13 (263/268)	97.28-98.72	100 (20/20)
*E. crandallis*	87.68 (235/268)	85.20-88.80	100(20/20)
*E. parva*	79.10 (212/268)	75.99-82.01	95 (19/20)
*E. bakuensis*	60.82 (163/268)	57.11-62.89	100(20/20)
*E. pallida*	34.32 (92/268)	31.23-36.77	75 (15/20)
*E. faurei*	33.58 (90/268)	30.11-35.89	90 (18/20)
*E. granulosa*	23.13 (62/268)	19.63-26.37	65 (13/20)
*E. ahsata*	21.26 (57/268)	18.35-23.65	90 (18/20)
*E. intricata*	11.19 (30/268)	9.20-12.04	60 (12/20)
*E. punctata*	1.86 (5/268)	0.44-2.44	25 (5/20)

* Samples with OoPG ≥ 1000;

** CI: confidential interval.

According to epidemiological analyses, all variables were significantly associated with *Eimeria* infection (Table 2). Sheep raised in semi-intensive and intensive rearing systems were 2.1 (CI 95% = 1.4–3.1; p<0.05) and 46.5 (CI 95% = 6.4–337.2; p<0.05), respectively, more likely to be infected than sheep raised in an extensive system. The presence of sheepfold, no rotation of pasture, mixed rearing, and gender (male) were all considered risk factors to eimeriosis. The age of the animal and the type of the floor also influence the infection. Sheep with the age of 0-6 months (OR = 14.2; CI 95% = 7.2–27.9; p<0.05) and 6-12 months (OR = 3.9; CI 95% = 2.5-6.1), and raised on dirt floor (OR = 7.5; CI 95% = 4.1–13.7; p<0.05) are more likely to be infected.

**Table 2:** Analysis of variables associated with *Eimeria* infection in sheep from northern Paraná, Brazil.

**Variables**	**Positive/ Total (%)**	**p-value**	**OR** * **(CI 95%)** **
**Production system**			
Extensive	276/ 374 (73.8)	-	1
Semi-intensive	243/ 284 (85.5)	0.0003	2.1 (1.4-3.1)
Intensive	131/132 (99.2)	0.0001	46.5 (6.4-337.2)
**Age**			
≤ 6	258/ 268 (96.2)	0.0001	14.2 (7.2-27.9)
6 ≤ 12	213/ 243 (87.6)	0.0001	3.9 (2.5-6.1)
> 12	191/296 (64.5)	-	1
**Rotation of pasture**			
No	553/ 659 (83.9)	0.0047	2.0 (1.2-2.9)
Yes	109/ 148 (73.6)		
**Sheepfold**			
Yes	546/ 651 (83.8)	0.0077	1.7 (1.1-2.17)
No	116/ 156 (74.3)		
**Floor**			
Dirt	439/ 502 (87.5)	0.0001	7.5 (4.1-13.7)
Slatted	82/ 97 (84.5)	0.0001	5.9 (2.7-12.8)
Wood shavings	25/ 27 (48.0)	-	1
**Mixed rearing**			
Yes	265/ 301 (88.0)	0.0008	2.0 (1.4-3.0)
No	397/ 506 (78.4)		
**Gender**			
Male	164/ 176 (93.2)	0.0001	3.6 (1.9-6.7)
Female	498/ 631 (78.9)		

* OR: *odds ratio*;

** CI: confidence interval.

## Discussion

We observed that 82.03% of sheep were infected with *Eimeria* spp. Similar results were found in previous studies, which showed a prevalence of 94.6% and 85.98% in sheep and lambs from the states of the Rio de Janeiro and the Rio Grande do Sul, respectively (Hassum & Menezes, 2005; Martins et al., 2020). Studies conducted worldwide have shown a prevalence of *Eimeria* infection ranging from 57.7% in Egypt to 92.9% in China (Wang et al., 2010; Mohamaden et al., 2018). The difference in prevalence depends on several factors, such as climate and management conditions, including weaning, changes in feed, transportation, and stress (Carrau et al., 2018).

In this study, lambs (aged <12 months) were more affected than adult sheep. Souza et al. (2015) also observed that young animals had a higher prevalence (68.2%) than adults (39.6%), indicating that age is one of the main factors influencing the occurrence of *Eimeria* infection in sheep. The high susceptibility of young animals is related to immunological aspects, with adults acquiring specific immunity against *Eimeria* spp. after initial exposure (Hermosilla et al., 2012). Thus, eimeriosis is considered a self-limiting disease. It has a progressive increase in the prevalence and intensity of oocyst shedding until it reaches a peak close to the weaning period and is reduced in adults (Silva et al., 2011; Chartier & Paraud, 2012).

Animals reared in semi-intensive and intensive systems had a statistically significant difference in infection rates compared to those reared in extensive systems. Previous studies have shown that the prevalence of *Eimeria* spp. in the intensive rearing system can reach up to 92.7% (Saratsis et al., 2011), while in the extensive rearing system, it ranges from 25.3% to 58.9% (Ahid et al., 2009; Brito et al., 2009). These studies indicate that *Eimeria* is present in all production systems; however, it has been considered more frequent in animals reared in intensive systems because of the high population density, which increases the number of oocysts shedding in the environment (Yakhchali & Golami, 2008; Lopes et al., 2013).

In this study, male animals were 3.6 times more likely to be infected by *Eimeria* spp. than female animals. The higher susceptibility of male animals to infections may be associated with immunosuppression caused by elevated plasma levels of androgens, especially testosterone, during the reproductive season (Bhat et al., 2012; Souza et al., 2015). However, a previous study reported lower positivity for *Eimeria* infection in males than in females, suggesting factors such as the physiological state of females and higher number of analyzed samples (Tembue et al., 2009). Further investigations are needed to investigate the effects of sex in eimeriosis in sheep.

Sheep raised with the sheepfold were 1.7 times more likely to have *Eimeria* than those raised without sheepfolds. The facilities and utensils used to raise sheep are important in the epidemiology of eimeriosis because they can be an important source of contamination (Chartier & Paraud, 2012). Previous reports indicate that sheep infections are related to poor hygienic conditions, including wet areas such as dirty and damp litter (Lopes et al., 2014).

Sheep kept on dirt floors had 7.5 times more chances of infection, whereas those sheep kept on slatted floors had 5.9 times compared to sheep kept on wood shavings, indicating that the use of wood shavings on the floor proved to be a protective factor against eimeriosis. Raising sheep in sheepfold with higher density and no management prophylaxis has been indicated as a risk factor for eimeriosis because it creates favorable conditions for oocyst sporulation (Andrews, 2013; Carrau et al., 2018).

We observed that sheep raised with other animal species, such as cattle, birds, horses, goats, had 2.0 times more chances of infection (p=0.0008). Moreover, farms that do not perform pasture rotation are 2.0 times more likely to have infected animals than farms that follow this management strategy. *Eimeria* species are species-specific organisms, and the high population density in mixed animal breeding facilities may cause stress and result in susceptibility to eimeriosis. Thus, the hygiene of the facilities and adequate management conditions are essential for reducing viable oocysts in the environment and controlling eimeriosis in sheep (Cai & Bai, 2009; Chartier & Paraud, 2012).

The most prevalent species in this study were *E. ovinoidalis* and *E. crandallis*. These species are commonly reported in sheep worldwide (Saratsis et al., 2011; Lopes et al., 2013; El-Alfy et al., 2020; Olmos et al., 2020; Al-Neama et al., 2021), and the predominance could be due to their higher reproductive efficiency compared to other species (Catchpole et al., 1976). *Eimeria ovinoidalis* and *E. crandallis* are pathogenic, and they are responsible for clinical coccidiosis, even in adult sheep (Olmos et al., 2020).

Most of the animals in our study were infected with different species, including pathogenic and non-pathogenic species. These multiple infections have already been described in small ruminants, where more than 80% of fecal samples have two or more species (Wang et al., 2010; Silva et al., 2011). A previous study showed that co-infection with a different species of *Eimeria* is a predictor of the probability of infection, indicating that this condition contributes to the epidemiology of eimeriosis in sheep (Al-Neama et al., 2021).

## Conclusions

A high prevalence of *Eimeria* natural infection was recorded in sheep from northern Paraná, Brazil. The analysis of risk factors showed that intensive rearing, no rotation of pasture, dirt and slatted floors, and age up to 12 months were associated with eimeriosis in sheep. Thus, good management and hygiene practices must be employed to avoid infection and economic losses.

## References

[B001] Ahid SMM, Medeiros VMC, Bezerra ACDS, Maia MB, Lima VXM, Vieira LS (2009). Espécie do gênero *Eimeria* Schneider, 1875 (*Apicomplexa: Eimeriidae*) em pequenos ruminantes na mesorregião oeste do estado do Rio Grande do Norte, Brasil. Cienc Anim Bras.

[B002] Al-Neama RT, Bown KJ, Blake DP, Birtles RJ (2021). Determinants of *Eimeria* and *Campylobacter* infection dynamics in UK domestic sheep: the role of co-infection. Parasitology.

[B003] Andrews AH (2013). Some aspects of coccidiosis in sheep and goats. Small Rumin Res.

[B004] Arslan MO, Umur S, Kara M (1999). The prevalence of coccidian species in sheep in Kars Province of Turkey. Trop Anim Health Prod.

[B005] Basiaga M, Levytska V, Kowal J, Nosal P, Wyrobisz-Papiewska A (2020). Coccidiosis: a problem in backyard rabbitries. Ann Parasitol.

[B006] Bhat S, Mir M, Qadir S, Allaie I, Khan H, Husain I (2012). Prevalence of gastro-intestinal parasitic infections in Sheep of Kashmir valley of India. Vet World.

[B007] Brito DRB, Santos ACG, Teixeira WC, Guerra RMSNC (2009). Parasitos gastrintestinais em caprinos e ovinos da microrregião do Alto Mearim e Grajaú, estado do Maranhão, Brasil. Cienc Anim Bras.

[B008] Bruhn FRP, Lopes MA, Demeu FA, Perazza CA, Pedrosa MF, Guimarães AM (2011). Frequency of species of *Eimeria* in females of the holstein-friesian breed at the post-weaning stage during autumn and winter. Rev Bras Parasitol Vet.

[B009] Cai KZ, Bai JL (2009). Infection intensity of gastrointestinal nematodosis and coccidiosis of sheep raised under three types of feeding and management regims in Ningxia Hui Autonomous Region, China. Small Rumin Res.

[B010] Cardim ST, Seixas M, Tabacow VBD, Taroda A, Carneiro PG, Martins TA (2018). Prevalence of *Eimeria* spp. in calves from dairy farms in northern Paraná state, Brazil. Rev Bras Parasitol Vet.

[B011] Carrau T, Silva LMR, Pérez D, Failing K, Martínez-Carrasco C, Macías J (2018). Associated risk factors influencing ovine *Eimeria* infections in southern Spain. Vet Parasitol.

[B012] Catchpole J, Norton CC, Joyner LP (1976). Experiments with defined multispecific coccidial infections in lambs. Parasitology.

[B013] Chartier C, Paraud C (2012). Coccidiosis due to *Eimeria* in sheep and goats, a review. Small Rumin Res.

[B014] Eckert J, Brown R, Shirley MW, Coudert P (1995). Biotechnology: guidelines on techniques in coccidiosis research..

[B015] El-Alfy E-S, Abbas I, Al-Kappany Y, Al-Araby M, Abu-Elwafa S, Dubey JP (2020). Prevalence of *Eimeria* species in sheep (*Ovis aries*) from Dakahlia governorate, Egypt. J Parasit Dis.

[B016] Fallis M (1974). Review of protozoan parasites of domestic animals and man, by N. D. Levine. J Parasitol.

[B017] Florião MM, Lopes BB, Berto BP, Lopes CWG (2016). New approaches for morphological diagnosis of bovine *Eimeria* species: a study on a subtropical organic dairy farm in Brazil. Trop Anim Health Prod.

[B018] Hassum IC, Menezes RCAA (2005). Infecção natural por espécies do gênero *Eimeria* em pequenos ruminantes criados em dois municípios do estado do Rio de Janeiro. Rev Bras Parasitol Vet.

[B019] Hermosilla C, Ruiz A, Taubert A (2012). *Eimeria bovis*: an update on parasite–host cell interactions. Int J Med Microbiol.

[B020] IAPAR (2018). Dados diários: agrometeorologia.

[B021] Keeton STN, Navarre CB (2018). Coccidiosis in large and small ruminants. Vet Clin North Am Food Anim Pract.

[B022] Lopes WDZ, Borges FA, Faiolla TP, Antunes LT, Borges DGL, Rodriguez FS (2013). *Eimeria* species in young and adult sheep raised under intensive and/or semi-intensive systems of a herd from Umuarama city, Parana State, Brazil. Cienc Rural.

[B023] Lopes WDZ, Carvalho RS, Pereira V, Martinez AC, Cruz BC, Teixeira WF (2014). Efficacy of sulfadoxine+trimethoprim compared to management measures for the control of *Eimeria* parasitism in naturally infected and clinically asymptomatic sheep that were maintained in a feedlot. Small Rumin Res.

[B024] Martins NS, Motta SP, Santos CC, Moreira AS, Farias NAR, Ruas JL (2020). *Eimeria* spp. infection in lambs from southern Brazil. Pesq Vet Bras.

[B025] Mohamaden WI, Sallam NH, Abouelhassan EM (2018). Prevalence of *Eimeria* species among sheep and goats in Suez Governorate, Egypt. Int J Vet Sci Med.

[B026] Olmos LH, Colque Caro LA, Avellaneda-Cáceres A, Medina DM, Sandoval V, Aguirre DH (2020). First record of clinical coccidiosis (*Eimeria ovinoidalis*) in adult sheep from northwestern Argentina. Vet Parasitol Reg Stud Rep.

[B027] Saratsis A, Joachim A, Alexandros S, Sotiraki S (2011). Lamb coccidiosis dynamics in different dairy production systems. Vet Parasitol.

[B028] Silva RM, Facury-Filho EJ, Souza MF, Ribeiro MFB (2011). Natural infection by *Eimeria* spp. in a cohort of lambs raised extensively in Northeast Brazil. Rev Bras Parasitol Vet.

[B029] Silva TP, Facury-Filho EJ, Nunes ABV, Albuquerque FHMAR, Ferreira PM, Carvalho AU (2007). Dinâmica da infecção natural por *Eimeria* spp. em cordeiros da raça Santa Inês criados em sistema semi-intensivo no Norte de Minas Gerais. Arq Bras Med Vet Zootec.

[B030] Souza LEB, Cruz JF, Teixeira MR, Albuquerque GR, Melo ADB, Tapia DMT (2015). Epidemiology of *Eimeria* infections in sheep raised extensively in a semiarid region of Brazil. Rev Bras Parasitol Vet.

[B031] Tembue AASM, Lima MM, Ramos RAN, Faustino MAG, Meunier IMJ, Alves LC (2009). Espécies do gênero *Eimeria* Schneider, 1875 (Apicomplexa: Eimeriidae) em pequenos ruminantes, provenientes do município de Ibimirim, estado de Pernambuco. Vet Not.

[B032] Ueno H, Gonçalves PC (1998). Manual para diagnóstico da helmintoses de ruminantes..

[B033] Wang CR, Xiao JY, Chen AH, Chen J, Wang Y, Gao JF (2010). Prevalence of coccidial infection in sheep and goats in northeastern China. Vet Parasitol.

[B034] Yakhchali M, Golami E (2008). *Eimeria* infection (Coccidia: Eimeriidae) in sheep of different age groups in Sanandaj city, Iran. Vet Arh.

